# Functional role of PPAR-γ on the proliferation and migration of fibroblast-like synoviocytes in rheumatoid arthritis

**DOI:** 10.1038/s41598-017-12570-6

**Published:** 2017-10-04

**Authors:** Xiao-Feng Li, Ying-Yin Sun, Jing Bao, Xin Chen, Yu-Huan Li, Yang Yang, Lei Zhang, Cheng Huang, Bao-Ming Wu, Xiao-Ming Meng, Jun Li

**Affiliations:** 1 School of Pharmacy, Anhui Medical University, Hefei, 230032 China; 2 Institute for Liver Diseases of Anhui Medical University (AMU), Anhui Medical University, Hefei, 230032 China; 3 Anhui Institute of Innovative Drugs, Hefei, 230032 China; 4 Hematology Department, The First Affiliated Hospital of Anhui Medical University, Hefei, 230022 China

## Abstract

Peroxisome proliferator-activated receptor (PPAR)-γ is involved in both normal physiological processes and pathology of various diseases. The purpose of this study was to explore the function and underlying mechanisms of PPAR-γ in rheumatoid arthritis (RA) fibroblast-like synoviocytes (FLSs) proliferation and migration. In the present study, we found PPAR-γ expression was remarkably reduced in RA synovium patient compare with OA and normal, as well as it was low-expression in Adjuvant-induced arthritis (AA). Moreover, inhibition PPAR-γ expression by T0070907 (12.5 μM) or PPAR-γ siRNA could promote FLSs proliferation and expressions of c-Myc, Cyclin D1, MMP-1, and MMP-9 in AA FLSs, except for TIPM-1. These date indicate that up-regulation of PPAR-γ may play a critical role in RA FLSs. Interestingly, co-incubation FLSs with Pioditazone (25 μM) and over expression vector with pEGFP-N1-PPAR-γ reduced proliferation and expressions of c-Myc, Cyclin D1, MMP-1, and MMP-9 in AA FLSs, besides TIMP-1. Further study indicates that PPAR-γ may induce activation Wnt/β-catenin signaling. In short, these results indicate that PPAR-γ may play a pivotal role during FLSs activation and activation of Wnt/β-catenin signaling pathway.

## Introduction

Rheumatoid arthritis (RA) is characterized by tumor-like expansion of the synovium and the subsequent destruction of adjacent articular cartilage and bone^1^. At the same time, angiogenesis is required to maintain the chronic inflammatory state by transporting inflammatory cells to the site of synovitis and supplying nutrients to the pannus^2^. In RA synovium, migration and invasion of activated fibroblast-like synoviocytes (FLSs), the major cell population in invasive pannus, actively participate in the inflammatory processes of RA^1,3^, such as produce pro-inflammatory cytokines TNF-α^3,4^ and IL-6^5,6^, matrix metalloproteinases and angiogenic factors^7^. One of the RA features is the excessive proliferation, migration and activation of FLSs and formation of pannus that invades adjacent cartilage and bone^8^. So inhibition of FLSs proliferation and migration is an ideal target for the treatment of RA. Many research studies^9,10^ have been carried out the expression of c-Myc, Cyclin D1, MMP-3, MMP-9 and TIMP-1are proved to be related with cell proliferation and migration. Although the exact causes of RA remain unknown, FLSs proliferation, migration and immunological dysregulation by inflammatory cytokines^1^ has been shown to be involved in driving the inflammation and synovial cell proliferation that result in joint destruction in RA patients. However, little is currently known on the molecular mechanisms underlying proliferation, migration and invasion of activated FLS.

The latest achievement obviously suggested that peroxisome proliferator-activated receptor (PPAR)-γ might contribute to the persistent expression of pro-inflammatory cytokines in RA^11,12^. The PPAR-γ belongs to the PPAR family of nuclear hormone receptors best known for their role in regulating various genes involved in glucose homeostasis, lipid metabolism, and adipocyte differentiation^13,14^. Pioglitazone, a thiazolidinedione class synthetic PPAR-γ agonist, is used for treatment of patients with type II diabetes mellitus^15^. More importantly, PPAR-γ has been known to have remarkably anti-inflammatory activities and anti-proliferation^16,17^. In a recent study have performed that PPAR-γ was significantly associated with RA^11,17,18^. However, it remains unknown the expresses of PPAR-γ and what is the pathophysiologic role of FLSs proliferation in RA.

An increasing body of evidence has indicated that Wnt/β-catenin pathway, one might rationalize that untimely activation of Wnt/β-catenin pathway is partly responsible for driving RA FLS activation and RA pathogenesis^19,20^. The Wnt/β-catenin pathway is a conserved signal transduction pathway that regulates a variety of biological processes, including signal transduction, cell cycle, cell proliferation, migration, differentiation, apoptosis, cell adhesion and tumorigenesis, and play a important role in limb development and joint formation^19,21,22^. In the absence of Wnt ligand, the signaling pathways in the resting state, β-catenin level is widely used as a sentinel marker Wnt/β-catenin pathway under pathological conditions^23,24^.

To further elucidate the relationship between PPAR-γ and FLSs activation in RA, in particularly, we found that the increased expression of PPAR-γ contributed to proliferation and migration of FLSs and was closely associated with Wnt/β-catenin pathway activation in RA pathogenesis.

## Results

### The expression of PPAR-γ was down-regulated in RA FLSs

**Figure 1:**
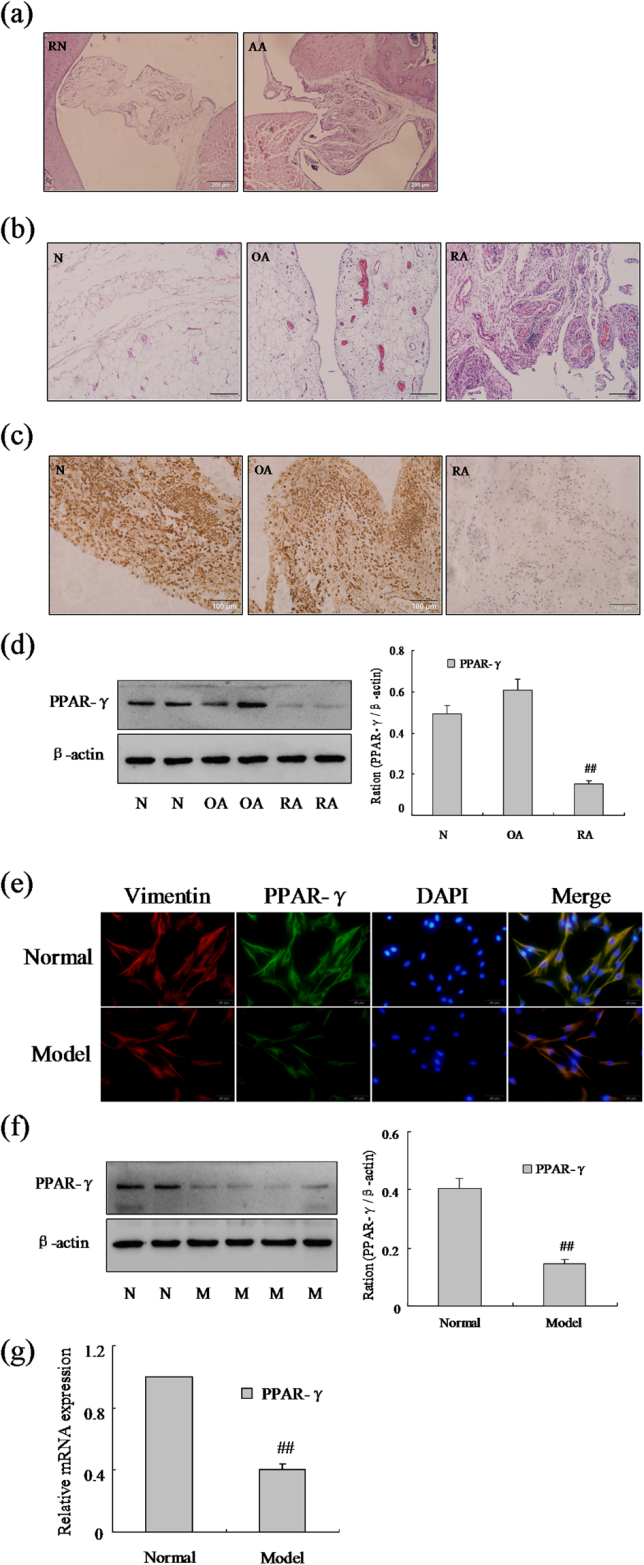
The expression of PPAR-γ was down-regulated in RA FLSs. (**a**) Representative H&E staining of AA and normal synovial tissues in rat (original magnification, ×10). (**b**) Representative H&E staining of RA, OA and normal synovial tissues in human (original magnification, ×10). (**c**) The expression of PPAR-γ in RA, OA and normal synovial tissue was analyzed by IHC staining analysis in human. (**d**) The protein level of PPAR-γ was analyzed by Western blot in RA, OA and normal synovial tissue. (**e**) The expression of PPAR-γ and Vimentin were analyzed by double immunofluorescence staining analysis in rat AA and normal FLSs. (**f**) The protein level of PPAR-γ was analyzed by Western blot in AA and normal FLSs. (**g**) The mRNA level of PPAR-γ was analyzed by Q-PCR in AA and normal FLSs. All values were expressed as mean ± SEM. ^##^P < 0.01 vs normal group.

To affirm the role of PPAR-γ in RA, model of AA was established by injection with the complete Freund’s adjuvant. Histopathological analysis (Fig. 1a) confirmed the model of AA was established successfully, increased remarkable inflammatory cells infiltrations. We performed immunohistochemical and western blot analysis the expression of PPAR-γ was down-regulated observably in RA FLSs compare with normal, as show Fig. 1b,c and d. The expression of Vimentin studies (Fig. 1e) indicated that the cells were derived from synovial tissues was FLSs. Similarly, western blot and Q-PCR analysis showed PPAR-γ mRNA and protein levels (Fig. 1f,g) were down-regulated significantly in FLSs isolated from AA rats’ synovium. Moreover, we also have measured PPAR-γ protein expression by immunofluorescence staining (Fig. 1e) in AA FLSs was lower than normal FLSs. Thus, these results suggesting that the expression of PPAR-γ was observably reduced in RA FLSs.

### Effect of PPAR-γ inhibitor increases FLSs proliferation and migration

**Figure 2:**
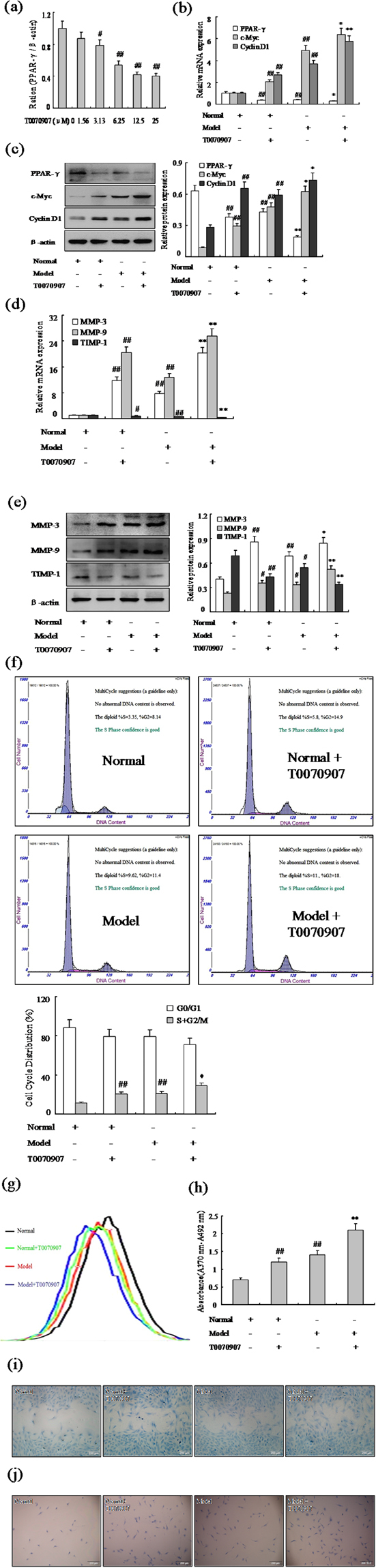
Effect of PPAR-γ inhibitor increases FLSs proliferation and migration. (**a**) Concentration-dependent inhibition expression of PPAR-γ mRNA by T0070907 in normal FLSs, tested by Q-PCR assays. (**b**) The mRNA levels of PPAR-γ, c-Myc and Cyclin D1 were analyzed by Q-PCR in FLSs with T0070907 (12.5 μM). (**c**) The protein levels of PPAR-γ, c-Myc and Cyclin D1 were analyzed by Western blot in FLSs with T0070907 (12.5 μM). (**d**) The mRNA levels of MMP-3, MMP-9 and TIMP-1 were analyzed by Q-PCR in FLSs with T0070907 (12.5 μM). (**e**) The protein levels of MMP-3, MMP-9 and TIMP-1 were analyzed by Western blot in FLSs with T0070907 (12.5 μM). (**f**) Cell cycle of FLSs were incubated with T0070907 (12.5 μM) for 48 h and then subjected to the FACS analysis. (**g**) After stained with CFDA-SE, FLSs were incubated with T0070907 (12.5 μM) for six days and then subjected to the FACS analysis. (**h**) BrdU proliferation assay were treated with T0070907 (12.5 μM) 48 h in FLSs. (**i**) FLSs were treated with T0070907 (12.5 μM), and migration into the wound-healing 24 h was photographed (original magnification, ×10). (**j**) FLSs were treated with T0070907 (12.5 μM), and transwell migration 48 h was photographed (original magnification, ×10). All values were expressed as mean ± SEM. ^#^P < 0.05, ^##^P < 0.01 vs normal group. *P < 0.05, **P < 0.01 vs model group.

To identify the significance of PPAR-γ in the RA FLSs, we have measured the effect of PPAR-γ on the proliferation and migration FLSs by treated with PPAR-γ inhibitor T0070907 in AA and normal rats. First, we investigated the effect of T0070907 on the expression of PPAR-γ (Fig. 2a), addition of 12.5 μM suppressed obviously the expression of PPAR-γ mRNA with Q-PCR assays in normal FLSs as show Fig. 2b and c. The results of western blot and Q-PCR showed the expression of PPAR-γ was down-regulated markedly in varying degrees with 12.5 μM T0070907 in normal and AA FLSs as show Fig. 2b and c. More significantly, western blot and Q-PCR has verified expression of protooncogene c-Myc and cyclin protein Cyclin D1 were up-regulated markedly by PPAR-γ inhibitor 12.5 μM T0070907 in normal and AA FLSs (Fig. 2b and c). In addition, MMP-3 and MMP-9 mRNA and protein expression also were up-regulated observably by T0070907 in normal and AA FLSs, whereas TIPM-1 was down-regulated (Fig. 2d and e). Similarly, cell cycle analysis (Fig. 2f) also suggested that treatment of FLSs with T0070907 resulted in increased varying degrees in S phase and G2/M phase. Remarkably, evidences were collected by CFDA SE cell proliferation assay and BrdU cell proliferation ELISA, as show Fig. 2b and c, that the normal and AA FLS were promoted observably proliferation with T0070907. What’s more, wound-healing assay and cell invasion assay have proved that the normal and AA FLS were promoted observably migration with T0070907 (Fig. 2i and j). Thus, these researches had proved that effect of PPAR-γ inhibitor T0070907 increased markedly FLSs proliferation and migration in normal and AA rats.

### Effect of PPAR-γ siRNA increases FLSs proliferation and migration

**Figure 3:**
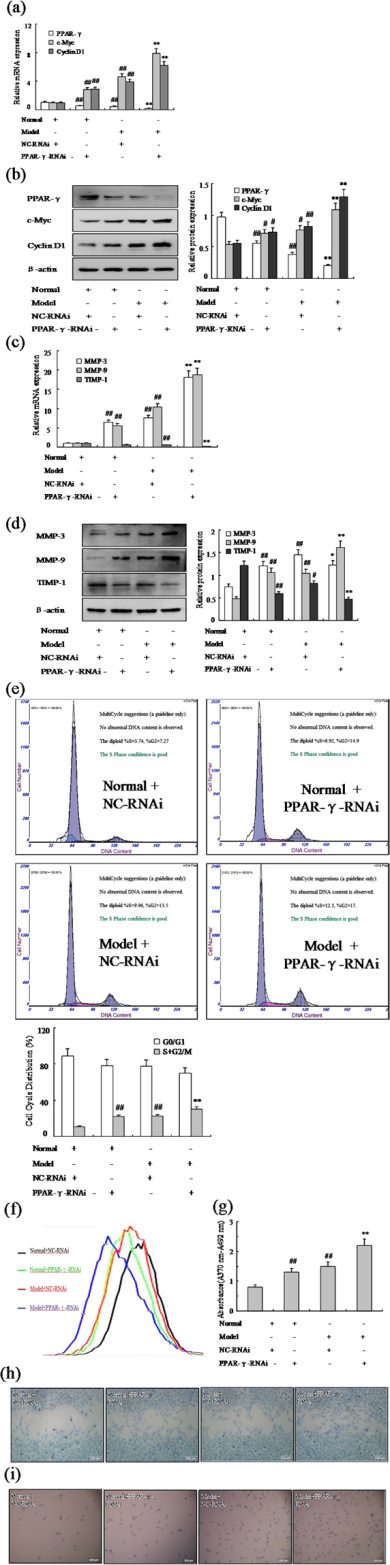
Effect of PPAR-γ siRNA silencing increases FLSs proliferation and migration. (**a**) The mRNA levels of PPAR-γ, c-Myc and Cyclin D1 were analyzed by Q-PCR in FLSs with PPAR-γ siRNA. (**b**) The protein levels of PPAR-γ, c-Myc and Cyclin D1 were analyzed by Western blot in FLSs with PPAR-γ siRNA. (**c**) The mRNA levels of MMP-3, MMP-9 and TIMP-1 were analyzed by Q-PCR in FLSs with PPAR-γ siRNA. (**d**) The protein levels of MMP-3, MMP-9 and TIMP-1 were analyzed by Western blot in FLSs with PPAR-γ siRNA. (**e**) Cell cycle of FLSs were incubated with PPAR-γ siRNA for 48 h and then subjected to the FACS analysis. (**f**) After stained with CFDA-SE, FLSs were incubated with PPAR-γ siRNA for six days and then subjected to the FACS analysis. (**g**) BrdU proliferation assay were treated with PPAR-γ siRNA 48 h in FLSs. (**h**) FLSs were treated with PPAR-γ siRNA, and migration into the wound-healing 24 h was photographed (original magnification, ×10). (**i**) FLSs were treated with PPAR-γ siRNA, and transwell migration 48 h was photographed (original magnification, ×10). All values were expressed as mean ± SEM. ^#^P < 0.05, ^##^P < 0.01 vs normal group. *P < 0.05, **P < 0.01 vs model group.

In order to provide additional evidence that PPAR-γ is involved in the proliferation and migration of RA FLSs, siRNA specific for rat PPAR-γ was used to knockdown gene expression in AA FLSs. Normal and AA FLSs treated with control siRNA or PPAR-γ siRNA was exposed to 48 h following the transfection with 100 nM PPAR-γ siRNA, the level of PPAR-γ mRNA and protein were reduced remarkably compared with the cells transfected with control siRNA (Fig. 3a). As same as expected, the expression of c-Myc and Cyclin D1 mRNA and protein were up-regulated by PPAR-γ siRNA in normal and AA FLSs as show Fig. 3a and b. Furthermore, MMP-3 and MMP-9 mRNA and protein expression also were up-regulated observably by PPAR-γ siRNA, however, TIPM-1 were down-regulated markedly in normal and AA FLSs as show Fig. 3c and d. Similarly, cell cycle analysis also suggested that treatment of FLSs with PPAR-γ siRNA resulted in increased varying degrees in S phase and G2/M phase in normal and AA rats (Fig. 3e). Remarkably, evidence was collected by CFDA SE cell proliferation assay and BrdU cell proliferation ELISA, as show Fig. 3f and g, that the normal and AA FLS were promoted observably proliferation with PPAR-γ siRNA. What’s more, wound-healing assay and cell invasion assay (Fig. 3h and i) have proved that the normal and AA FLS were promoted observably migration with PPAR-γ siRNA. Taken together, our findings suggested that inhibited PPAR-γ expression could increase FLSs proliferation and migration in normal and AA rats and PPAR-γ may contribute to the progression of RA.

### Effect of PPAR-γ agonist inhibits FLSs proliferation and migration

**Figure 4:**
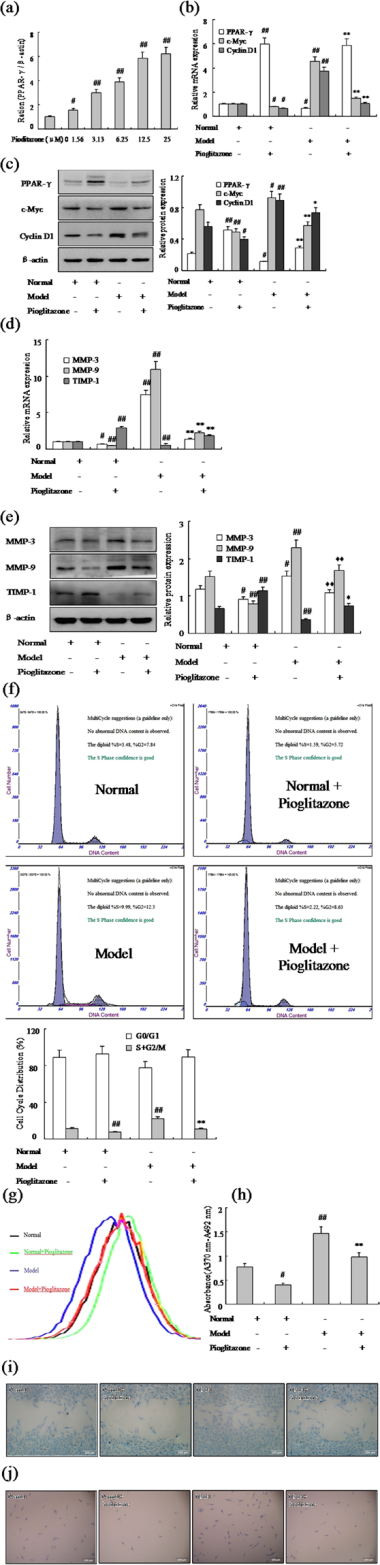
Effect of PPAR-γ agonist inhibits FLSs proliferation and migration. (**a**) Concentration-dependent inhibition expression of PPAR-γ mRNA by Pioditazone in AA FLSs, tested by Q-PCR assays. (**b**) The mRNA levels of PPAR-γ, c-Myc and Cyclin D1 were analyzed by Q-PCR in FLSs with Pioditazone. (**c**) The protein levels of PPAR-γ, c-Myc and Cyclin D1 were analyzed by Western blot in FLSs with Pioditazone. (**d**) The mRNA levels of MMP-3, MMP-9 and TIMP-1 were analyzed by Q-PCR in FLSs with Pioditazone. (**e**) The protein levels of MMP-3, MMP-9 and TIMP-1 were analyzed by Western blot in FLSs with Pioditazone. (**f**) Cell cycle of FLSs were incubated with Pioditazone for 48 h and then subjected to the FACS analysis. (**g**) After stained with CFDA-SE, FLSs were incubated with Pioditazone for six days and then subjected to the FACS analysis. (**h**) BrdU proliferation assay were treated with Pioditazone 48 h in FLSs. (**i**) FLSs were treated with Pioditazone, and migration into the wound-healing 24 h was photographed (original magnification, ×10). (**j**) FLSs were treated with Pioditazone, and transwell migration 48 h was photographed (original magnification, ×10). All values were expressed as mean ± SEM. ^#^P < 0.05, ^##^P < 0.01 vs normal group. *P < 0.05, **P < 0.01 vs model group.

This is so clear that inhibited PPAR-γ expression could increase FLSs proliferation and migration in normal and AA rats. Based on the above research, we proposed over-expression of PPAR-γ may inhibit FLSs proliferation and migration. To further determine the underlying mechanism of PPAR-γ during FLSs proliferation and migration, PPAR-γ agonist Pioditazone hydrochloride was used to over-expression of PPAR-γ. Q-PCR assays showed 25 μM Pioditazone could increase obviously the expression of PPAR-γ mRNA in AA FLSs (Fig. 4a). In addition, with 25 μM Pioditazone in FLSs, the expression of PPAR-γ was up-regulated significantly in normal and AA FLSs (Fig. 4b and c). Conversely, after treating with 25 μM Pioditazone, expressions of c-Myc and Cyclin D1 mRNA and protein were down-regulated remarkably in normal and AA FLSs, as show Fig. 4b and c. Meanwhile, MMP-3 and MMP-9 mRNA and protein expression (Fig. 4d and e) also were down-regulated observably by Pioditazone, whereas TIPM-1 were up-regulated markedly in normal and AA FLSs. Consistent with the cell proliferation results, as same as expected flow cytometry analysis showed over-expression of PPAR-γ by 25 μM Pioditazone for 48 h resulted in decreased in S phase and G2/M phase significantly in normal and AA rats, as show Fig. 4f. Remarkably, evidence was collected by CFDA SE cell proliferation assay and BrdU cell proliferation ELISA (Fig. 4g and h) that the normal and AA FLS were suppressed observably proliferation with Pioditazone. What’s more, wound-healing assay and cell invasion assay have proved that the normal and AA FLS were suppressed observably proliferation with Pioditazone, as show Fig. 4i and j. These results suggested that treatment of FLSs with Pio 25 μM had a profound inhibitory effect on proliferation and migration of FLSs.

### Effect of over expression vector of PPAR-γ inhibits FLSs proliferation and migration

**Figure 5:**
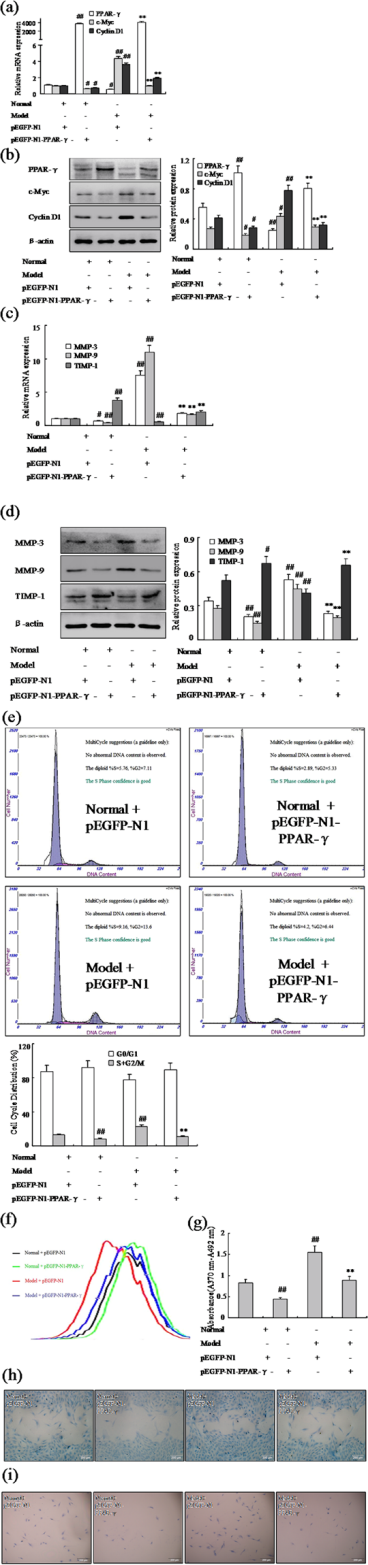
Effect of over expression vector of PPAR-γ inhibits FLSs proliferation and migration. (**a**) The mRNA levels of PPAR-γ, c-Myc and Cyclin D1 were analyzed by Q-PCR in FLSs with pEGFP-N1-PPAR-γ. (**b**) The protein levels of PPAR-γ, c-Myc and Cyclin D1 were analyzed by Western blot in FLSs with pEGFP-N1-PPAR-γ. (**c**) The mRNA levels of MMP-3, MMP-9 and TIMP-1 were analyzed by Q-PCR in FLSs with pEGFP-N1-PPAR-γ. (**d**) The protein levels of MMP-3, MMP-9 and TIMP-1 were analyzed by Western blot in FLSs with pEGFP-N1-PPAR-γ. (**e**) Cell cycle of FLSs were incubated with pEGFP-N1-PPAR-γ for 48 h and then subjected to the FACS analysis. (**f**) After stained with CFDA-SE, FLSs were incubated with pEGFP-N1-PPAR-γ for six days and then subjected to the FACS analysis. (**g**) BrdU proliferation assay were treated with pEGFP-N1-PPAR-γ 48 h in FLSs. (**h**) FLSs were treated with pEGFP-N1-PPAR-γ, and migration into the wound-healing 24 h was photographed (original magnification, ×10). (**i**) FLSs were treated with pEGFP-N1-PPAR-γ, and transwell migration 48 h was photographed (original magnification, ×10). All values were expressed as mean ± SEM. ^#^P < 0.05, ^##^P < 0.01 vs normal group. *P < 0.05, **P < 0.01 vs model group.

In order to provide additional evidence that PPAR-γ is involved in the proliferation and migration of RA FLSs, over expression vector with pEGFP-N1-PPAR-γ for rat was used to over expression of PPAR-γ in AA FLSs. Western blot and Q-PCR (Fig. 5a and b) showed the expression of PPAR-γ was up-regulated observably with pEGFP-N1-PPAR-γ in normal and AA FLSs. In addition, after treating with pEGFP-N1-PPAR-γ, expressions of c-Myc and Cyclin D1 mRNA and protein were down-regulated remarkably in normal and AA FLSs (Fig. 5a and b). Meanwhile, MMP-3 and MMP-9 mRNA and protein expression also were down-regulated observably by pEGFP-N1-PPAR-γ, conversely, TIPM-1 were up-regulated markedly, as show Fig. 5c and d. As same as Pioditazone, over-expression of PPAR-γ by pEGFP-N1-PPAR-γ resulted in decreased in S phase and G2/M phase significantly by flow cytometry analysis in normal and AA rats (Fig. 5e). Consistent with the cell proliferation results, CFDA SE cell proliferation assay and BrdU cell proliferation ELISA (Fig. 5f and g) has proved that the normal and AA FLS were suppressed observably proliferation with pEGFP-N1-PPAR-γ. What’s more, wound-healing assay and cell invasion assay have proved that the normal and AA FLS were suppressed observably proliferation with pEGFP-N1-PPAR-γ (Fig. 5h and i). These evidences proved that treatment of FLSs with pEGFP-N1-PPAR-γ had a profound inhibitory effect on proliferation and migration of FLSs. It may be involved in the proliferation of FLSs, and play a pivotal role in the pathogenesis of RA.

### PPAR-γ may modulate FLSs proliferation and migration and be closely associated with Wnt/β-catenin signaling pathway

**Figure 6:**
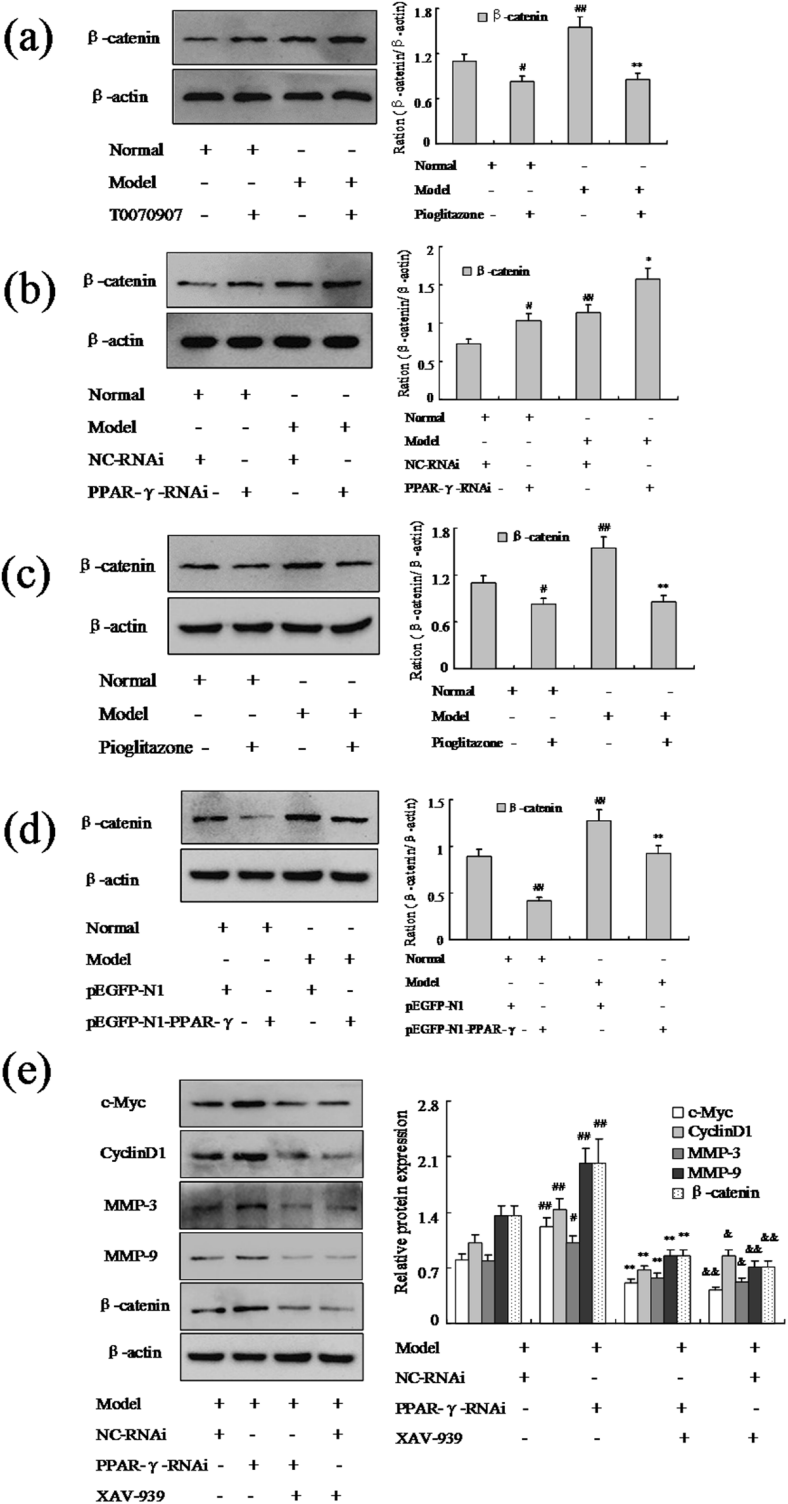
PPAR-γ may modulate FLSs proliferation and be closely associated with Wnt/β-catenin signaling pathway. (**a**) The protein level of β-catenin was analyzed by Western blot in FLSs with T0070907 (12.5 μM). (**b**) The protein level of β-catenin was analyzed by Western blot in FLSs with PPAR-γ siRNA. (**c**) The protein level of β-catenin was analyzed by Western blot in FLSs with Pioditazone (25 μM). (**d**) The protein level of β-catenin was analyzed by Western blot in FLSs with over expression vector pEGFP-N1-PPAR-γ. (**e**) The protein level of c-Myc, Cyclin D1, MMP-3, MMP-9 and β-catenin was analyzed by Western blot in AA FLSs. All values were expressed as mean ± SEM. ^#^P < 0.05, ^##^P < 0.01 vs normal group. *P < 0.05, **P < 0.01 vs model group. (**e**) ^#^P < 0.05, ^##^P < 0.01 vs model group; **P < 0.01 vs PPAR-γ siRNA group; ^&^P < 0.05, ^&&^P < 0.01 vs model group.

It has proved Wnt/β-catenin signaling plays a fundamental role in cell differentiation and proliferation. To investigate the effect of PPAR-γ on Wnt/β-catenin signaling in FLSs proliferation and migration, we firstly examined the expression profiles of β-catenin, a major component of Wnt/β-catenin pathway. Western blot analysis performed that β-catenin was up-regulated obviously in AA FLSs compare with normal. Moreover, inhibition PPAR-γ by T0070907 (12.5 μM) or PPAR-γ siRNA (Fig. 6a and b), β-catenin expression was inhanced significantly in normal and AA FLSs. In particular, Pioditazone (25 μM) or pEGFP-N1-PPAR-γ (Fig. 6c and d) supressed Wnt/β-catenin signaling, the expression of β-catenin was decreased substantially in normal and AA FLSs. In addition, Fig. 6e showed that after treating with PPAR-γ siRNA and XAV-939, expressions of c-Myc, Cyclin D1, MMP-3 and MMP-9 proteins were down-regulated remarkably, however, they were unchanged between with XAV-939 in AA FLSs. Taken together, all the above results indicated that PPAR-γ could modulate FLSs proliferation and migration and be closely associated with Wnt/β-catenin signaling pathway.

## Discussion

The results of this study show that: 1) the expression of PPAR-γ was down-regulated substantially in RA FLSs; 2) PPAR-γ inhibitor T0070907 or PPAR-γ siRNA could increase observably FLSs proliferation and migration in normal and AA; 3) PPAR-γ agonist Pioditazone or pEGFP-N1-PPAR-γ could suppress substantially FLSs proliferation and migration in normal and AA; 4) PPAR-γ could modulate FLSs proliferation and migration and be closely associated with Wnt/β-catenin signaling pathway.

Adjuvant arthritis (AA) is a model of experimental RA that is induced by injection complete Freund’s adjuvant (CFA)^25,26^. The AA in rats has similar characteristics to RA in aspects of histology and immunology and is a useful test system for evaluating therapies for RA. Therefore, we chose AA rats to affirm the role of PPAR-γ rather than RA. Recent progress in research has substantiated FLSs are key effector cells in inflammatory arthritic diseases^1,5^. In addition, the hyperplastic FLSs population potentially promotes lymphocyte and macrophage infiltration^27^, recruitment and retention by produces cytokines^28^, chemokines^29^, extracellular matrix proteins^27^ and cell adhesion molecules^30^. Migration and invasion of activated FLSs into cartilage and bone are critical events during invasive pannus formation in RA synovium^31^. However, there are no approved drugs that are known to target the FLSs in RA, and the underlying mechanisms driving FLSs activation remain unresolved. Hence, targeted suppress proliferation and migration of FLSs may potentially complement the current therapeutics without major deleterious effects on adaptive immune responses.

PPAR-γ is the most ample PPAR subtype in white adipose tissue, and its main function is administer of insulin sensitivity, fatty acid uptake and glucose homeostasis^32^. First, in this paper, we agree with Marder W and his colleagues^18^ that PPAR-γ is observably reduced by immunohistochemical and western blot in RA and AA FLSs compare with normal. Moreover, we also have measured PPAR-γ expression by immunofluorescence staining in AA FLSs was lower than normal. What’s more, inhibition of PPAR-γ expression with T0070907 12.5 μM or PPAR-γ siRNA could active protooncogene c-Myc, cyclin protein Cyclin D1, matrix metalloproteinases (MMPs) proteins MMP-3 and MMP-9 over-expression, whereas TIPM-1 was down-regulated. It is known that Cyclin D1 and c-Myc are common downstream molecules of this pathway, which are proved to be related with cell proliferation^33^. In addition, numerous experimental and clinical studies indicating the expression of MMP-3 and MMP-9 a family of zinc-dependent endopeptidases, provide condition for the cell migration and invasion^34^. On the other hand, the action of all of MMPs is regulated by a group of endogenous tissue inhibitors of metalloproteinases (TIMPs). More significantly, cell cycle analysis and CFDA SE cell proliferation assay, BrdU cell proliferation ELISA, wound-healing assay and cell invasion assay also suggested that inhibition of PPAR-γ expression increased markedly FLSs proliferation and migration in normal and AA rats.

It is necessary to consider whether over-expression of PPAR-γ could improve or regulate FLSs proliferation and migration. Indeed, PPAR-γ has been regarded as the receptor of the thiazolidinedione, a resultant chemical among anti-diabetic drugs, and in consequence, it could be targeted by many drug candidates^35^. Although not explicitly demonstrated, it is very likely that the side effects result from high doses of full agonists, such as Pioglitazone^36^, Bezafibrate^37^ and metaglidasen^38^. As a result, with treatment of FLSs with Pioglitazone 25 μM or over expression vector with pEGFP-N1-PPAR-γ had a profound inhibitory effect on proliferation and migration of FLSs in normal and AA rats. More importantly, PPAR-γ has been known to have remarkably anti-inflammatory activities^16,18^. In this study, our results showed that over-expression PPAR-γ could down-regulation expression of c-Myc, Cyclin D1, MMP-3 and MMP-9, and up-regulation expression of TIPM-1. To summarize, over-expression of PPAR-γ expression reduced significantly proliferation and migration of FLSs in AA. Regarding this last comment, of course, we suppose that PPAR-γ inhibits FLSs proliferation and migration through reduction insulin resistance and lipid metabolism, and further studies are required to comprehensively explore the role of PPAR-γ in RA.

This is so clear that activation of Wnt signaling disrupts this destruction complex, which leads to accumulation of β-catenin in the cytoplasm and finally translocation to nucleus. Our results also demonstrate this viewpoint that inhibition PPAR-γ by T0070907 12.5 μM or PPAR-γ siRNA, β-catenin expression was inhanced significantly in normal and AA FLSs. In particular, Pioglitazone 25 μM or pEGFP-N1-PPAR-γ supressed Wnt/β-catenin signaling, the expression of β-catenin was substantially decreased in normal and AA FLSs. Take together, these dates suggested that PPAR-γ mediated FLSs activation, proliferation and migration and was closely associated with activation of Wnt/β-catenin signaling pathway in RA.

In summary, our findings in the present study suggested that PPAR-γ might play a pivotal role during FLS activation and be closely associated with activation of Wnt/β-catenin signaling pathway. It is one more competent and qualified opinion that the over-expression of PPAR-γ suppresses AA FLSs proliferation and migration, indicating the potential of PPAR-γ as a therapeutic target for RA. Further research is needed to clarify if PPAR-γ could be used as a diagnostic marker and prognostic indicators of RA.

## Materials and Methods

### Human synovial tissue collection

Synovial tissue was obtained from patients with RA (n = 5) and osteoarthritis (OA, n = 15) according to the American College of Rheumatology 1987 revised criteria during joint synovectomies and 4 trauma patients with no history of acute or chronic arthritis served as controls. All patients signed informed consent to take part in the study. The study protocol was approved by the ethics boards of Anhui Medical University, and tissue specimen acquisition was performed in accordance with the institutional guidelines. The written informed consent was obtained from all subjects.

### Materials and reagents

T0070907 and Cell Proliferation ELISA, BrdU(colorimetric) were purchased from Sigma Inc. (St. Louis, MO, USA). Pioditazone hydrochloride (HPLC ≥ 98%) was purchased from Dalian Meilun Biotech Co., Ltd (Dalian, China). Rabbit anti-PPAR-γ, anti-β-catenin, anti-c-Myc and rabbit anti-Cyclin D1 monoclonal antibody were purchased from Cell Signaling (Danvers, MA, USA) and Rabbit anti-β-actin monoclonal antibody was purchased from Bioworld (Shanghai, China). Secondary antibody for goat anti-rabbit immunoglobulin (IgG) horse radish peroxidase (HRP) was purchased from Beijing Zhongshan Biotechnology Corporation (Beijing, China). PPAR-γ and β-actin primers were produced from Shanghai Sangon Biological and Technological Company (Shanghai, China).

### RA rat model

Adult female Sprague-Dawley (SD) rats (160–200 g) were treatment with complete Freund’s adjuvant (Sigma Inc., MO, USA) for 24 days 0.1 ml per 100 g body weight by paw injection at the left paw to induce RA rat models. Normal control rats were injected in paw with 0.1 ml of normal saline per 100 g body weight at same time. All the animal experiments were performed in accordance with the Regulations of the Experimental Animal Administration issued by the State Committee of Science and Technology of China. Efforts were made to minimize the number of animals used and their suffering. Animals were maintained in accordance with the Guides of Center for Developmental Biology, Anhui Medical University for the Care and Use of Laboratory. Animals and all experiments used protocols approved by the institutions’ subcommittees on animal care.

### Histopathology

The synovium specimens were fixed with 4% paraformaldehyde for 24 h and embedded in paraffin. Hematoxylin and eosin (H&E) staining and immunohistochemistry were performed according to a standard procedure. The pathological changes were assessed and photographed under an Olympus BX-51 microscope.

### Cell culture

FLSs were derived from the synovial tissues of AA and control rats. The cells were cultured in cell culture flasks in high-glucose DMEM medium (Hyclone, USA) supplemented with 20% (v/v) heat-inactivated fetal bovine serum (FBS) (Millipore, USA), 100 U/ml of penicillin, and 100 mg/ml of streptomycin (both from Beyotime, China). Cell cultures were maintained at 37 °C at an atmosphere of 5% CO_2_.

### Immunofluorescence staining

Cultured FLSs were plated in DMEM supplemented with 20% FBS at a density of 1–2 × 10^5^ cells/ml. Immunofluorescence staining was performed with Rabbit anti-PPAR-γ and anti-Vimentin (Alexa Fluor 594 Conjugated) (both from Cell Signaling, USA). Alexa Fluor 488-Conjugated AffiniPure Goat anti-rabbit IgG (H + L) (Beijing Zhongshan Biotechnology Corporation, China) was used as secondary antibody. Counterstaining of nuclei was performed with 4′,6-diamidino-2- phenylindole (DAPI; Beyotime, China).

### Small interfering RNA silencing

FLSs were transfected with 100 nM of small interfering RNA (siRNA) and pEGFP-N1-PPAR-γ using Lipofectamine 2000 (Invitrogen, CA, USA) according to the manufacturer’s instructions. The oligonucleotide sequences were as follows: PPAR-γ-siRNA (rat), 5′-CCUCCCUGAUGAAUAAAGATT-3′ for the sense strand and 5′-UCUUUAUUC AUCAGGGAGGTT-3′ for the antisense strand. A negative scrambled siRNA (GenePharma, Shanghai, China) was used in parallel. Cells were cultured at 37 °C for 6 h, and then, Q-PCR, Western blot, and flow cytometer (FCM) were used 48 h after siRNA transfection.

### Plasmid construction

Expression plasmid for PPAR-γ was generated by amplifying complementary DNA (cDNA) from pancreas cDNA and inserting cDNA coding for PPAR-γ into destination vectors by Gateway cloning (Invitrogen, USA). The N-terminal region of the PPAR-γ coding region containing the predicted CARD domain was cloned into pEGFP-N1 vector by using restriction sites SalI and AgeI. Cells transfection was performed with the Lipofectamine^TM^ 2000 according to the manufacturer’s manuals.

### Cell cycle analysis

To analyze the intracellular DNA content, FLSs were fixed in 70% ethanol at 4 °C overnight after 48 h treatment with T0070907, PPAR-γ-siRNA and Pioditazone, pEGFP-N1-PPAR-γ. FLSs were centrifuged at 1000 g for 5 min and re-suspended in PBS. After then, cells were stained with 0.5 ml of propidium iodide (PI) staining buffer (Beyotime, China), which contains 200 mg/ml RNase A, 50 μg/ml PI, at room temperature for 30 min in the dark. Flow cytometric analysis (FACS) was performed on Beckman Coulter.

### CFDA SE cell proliferation assay

Cell proliferation determination was conducted by CFDA SE probe (Beyotime, China). Briefly, cells (5 × 10^2^) were seeded and stained with CFDA-SE in 6-well plates according to the manufacturer’s protocol. Then, cells were exposed to T0070907, PPAR-γ-siRNA and Pioditazone, pEGFP-N1-PPAR-γ for six days. CFDA-SE fluorescence was detected by FACS.

### BrdU proliferation assay

FLSs (1000 cells/well) were seeded in 96-well plates with DMEM and 20% FBS and cultured 2 days. And then, Then, cells were exposed to T0070907, PPAR-γ-siRNA and Pioditazone, pEGFP-N1-PPAR-γ for48 h. The cells were labeled with 20 ml/well of BrdU labeling solution as described previously, and then incubated with 200 ml/well of FixDenat. After incubated with 100 ml/well of Anti-BrdU-POD working solution for 90 min and washed 3 times, substrate solution (TMB) was added and the absorbance of each well was read at 370 nm with an ELISA plate reader (Biotek, USA).

### Wound-healing

FLSs were with DMEM and 20% FBS and cultured 2 days in 24-well plate (5 × 10^5^/ml cells/well). And then, cells were serum deprived and scratched, treated with T0070907, PPAR-γ-siRNA and Pioditazone, pEGFP-N1-PPAR-γ. 24 h later Type II alveolar epithelial cells were fixed with methanol, stained with crystal violet stain, and viewed under an Olympus BX-51 microscope.

### Transwell experiments

For the migration assays, after with T0070907, PPAR-γ-siRNA and Pioditazone, pEGFP-N1-PPAR-γ, 1 × 10^5^ cells in 1% FBS media were placed into the upper chamber of an insert (8-μm pore size; Millipore, USA). And medium containing 20% FBS was added to the lower chamber. After incubation for 48 h, Cells that had migrated or invaded through the membrane were stained with methanol and 0.1% crystal violet stain, and viewed under an Olympus BX-51 microscope.

### Quantitative real-time PCR (Q-PCR)

Total RNA was extracted from cultured FLS by using TRIZol (Invitrogen, USA) according to the manufacture’s protocol, and reverse transcribed to cDNA using TAKARA kit (Japanese). The reaction mixture was prepared according to the manufacture’s instruction using SYBRGreen q-PCR Master Mix (Vazyme Biotech, Nanjing, China). The mRNA expression of PPAR-γ and β-actin were detected by the quantitative real-time PCR (q-PCR). The primers used were listed as following: PPAR-γ (forward: 5′-GCAAAGCAGAGACATCAGAAAG-3′; reverse: 5′-AGGTG GGGTCATCATACATAGG-3′), c-Myc (forward: 5′-TGCTCTCCGTCCTATGTTGC G-3′; reverse: 5′-CAGTCCTGGATGATGATGTTCTTGA-3′), Cyclin D1 (forward: 5′-CAGCGGTAGGGATGAAATAGTGA-3′; reverse: 5′-GGAATGGTTTTGGAAC ATGGAGA-3′), MMP-3 (forward: 5′-ATGATGAACGATGGACAGATGA-3′; reverse: 5′-CATTGGCTGAGTGAAAGAGACC-3′), MMP-9 (forward: 5′-CACT GTAACTGGGGGCAACT-3′; reverse: 5′-CACTTCTTGTCAGCGTCGAA-3′), TIMP-1 (forward: 5′-CATCTCTGGCC TCTGGCATC-3′; reverse: 5′-CATAACGCT GGTATAAGGTGGTCTC-3′), β-actin (forward: 5′-CCCATCTATGAGGGTTACGC -3′; reverse: 5′-TTTAATGTCACGCACGATTTC-3′). PCR was performed 95 °C for 10 minutes followed by 40 cycles at 95 °C for 15 seconds and at 60 °C about 1 minute by using Thermo Step One. Reactions were conducted three times, and threshold cycle values were normalized to β-actin gene expression. The specificity of the products was determined by melting curve analysis. Relative mRNA expression of target genes were obtained by normalized to control group and the level of β-actin.

### Western blot

Cultured FLSs were lysed with lysis buffer for Western (Beyotime, China). The whole-cell extracts (20 mg of protein) were then fractionated by electrophoresis through a 10–12% sodium dodecyl sulfate-polyacrylamide gel electrophoresis (SDS-PAGE) and blotted onto PVDF membranes (Millipore, USA). After blockade of nonspecific protein binding, nitrocellulose blots were incubated for more 12 h with primary antibodies diluted in primary antibody Dilution Buffer (Beyotime, China). Rabbit antibodies β-catenin, C-myc and CyclinD1 were used at 1:500 and β-actin was used at 1:1000. After incubation with primary antibodies, blots were washed four times in TBS/Tween-20 before incubation for 1 h in goat anti-mouse or anti-rabbit horse radish peroxidase conjugate antibody at 1:10000 dilutions in TBS/Tween-20 containing 5% skim milk. After washing four times with TBS/Tween-20 the protein blots were detected using the ECL-chemiluminescent kit (ECL-plus, Thermo Scientific, USA).

### Statistical analysis

Data are presented as means ± SD and were analyzed using SPSS16.0 software. Statistical significances were determined by one-way ANOVA with the post-hoc Dunnett’s test. In all cases, values of P < 0.05 were considered to be statistically significant.
